# Decades of Change in Vascular Plant Composition in High‐Latitude Ecosystems: Shifting Prevalence of Pollination Strategies

**DOI:** 10.1002/ece3.72288

**Published:** 2025-10-15

**Authors:** Petteri Kiilunen, Tuija Maliniemi, Janne Alahuhta, John‐Arvid Grytnes, Risto Virtanen, Kari Anne Bråthen, Konsta Happonen, Jutta Kapfer, Lauralotta Muurinen, Maria W. Skalska‐Tuomi, Terhi Ala‐Hulkko

**Affiliations:** ^1^ Geography Research Unit University of Oulu Oulu Finland; ^2^ Department of Biological Sciences University of Bergen Bergen Norway; ^3^ Faculty of Environmental Sciences and Natural Resource Management Norwegian University of Life Sciences Ås Norway; ^4^ Ecology and Genetics Research Unit University of Oulu Oulu Finland; ^5^ Department of Arctic and Marine Biology The Arctic University of Norway Tromsø Norway; ^6^ Finnish Youth Research Society Helsinki Finland; ^7^ Norwegian Institute of Bioeconomy Research Tromsø Norway; ^8^ Spatial Planning and Transportation Engineering: Department of Built Environment Aalto University Espoo Finland; ^9^ Kerttu Saalasti Institute, University of Oulu Nivala Finland

**Keywords:** boreal forests, ecosystem functioning, nectar, pollination, tundra, vegetation resurvey

## Abstract

The composition of high‐latitude plant communities has changed over the past decades in response to several global change drivers. However, less is known about how these compositional long‐term changes are reflected in the total cover of plant species that do or do not interact with pollinators. Using species‐specific indicator values for pollinator dependence and nectar production, we provide empirical evidence on how compositional changes in vascular plant communities over the past 50 years are reflected in the cover of pollinator‐dependent and pollinator‐independent plants, as well as the cover of pollen‐ and nectar‐rewarding and non‐nectar plants, in two ecosystems in northern Fennoscandia. We show that the average cover of pollinator‐independent plants greatly increased in both tundra and herb‐rich forest communities over time. Average cover of pollinator‐dependent plants slightly increased in tundra but decreased in herb‐rich forests. The average cover of pollen‐nectar plants increased in the tundra but decreased in herb‐rich forests over time. At the same time, the cover of non‐nectar plants increased in both ecosystems. The observed changes were strongly driven by the increased cover of evergreen dwarf shrubs in the tundra and the decline of forb cover in herb‐rich forests. The observed changes were comparable between sites that had been disturbed by human land use and sites that remained in a natural or semi‐natural state. Our results suggest that, in terms of average plant coverage, high‐latitude plant communities have broadly become less dependent on insect pollination over the past 50 years. By documenting long‐term changes in the pollination strategies of high‐latitude plant communities, our study underscores the need to explore how shifts in plant community composition are linked to pollination processes and broader plant–pollinator dynamics. We highlight patterns that warrant further investigation and offer perspectives for future research on plant–pollinator interactions in northern ecosystems under global change.

## Graphical abstract

We studied long‐term changes in plant species that require or support pollinators in comparison to those that do not interact with pollinators in two high‐latitude ecosystems of northern Fennoscandia. Over the past 50 years, plant communities in tundra and boreal herb‐rich forests have shifted toward reduced dependence on insect pollination, with pollinator‐independent and non‐nectar plants increasing in cover across both ecosystems. These changes were largely driven by the expansion of evergreen dwarf shrubs in tundra and the decline of forbs in forests and occurred regardless of human land use disturbance.

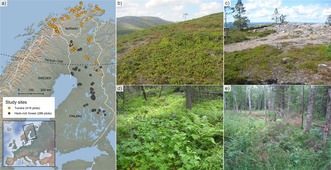

## 1 Introduction

Plants interact closely with other species in both natural and anthropogenic ecosystems (Scherber et al. 2010; Isbell et al. 2011), forming foundational relationships that regulate key ecosystem functions and services (MA 2005; IPBES 2019). For example, pollinators visit plant individuals in search of nectar and pollen as food, pollinating the plants in the process. This mutualistic partnership maintains plant communities through facilitating plant reproduction while simultaneously shaping pollinator communities by providing foraging resources (Potts et al. 2003; Nielsen et al. 2012). However, plant communities are dynamic, and their compositions are changing both naturally and because of human impacts. Currently, there is limited evidence on how long‐term changes in the composition of plant communities are reflected in the cover or abundance of pollinator‐dependent and pollinator‐independent plants (Biesmeijer et al. 2006; Ehlers et al. 2021; Pan et al. 2024) and plants that provide floral rewards for pollinators (Wallisdevries et al. 2012; Baude et al. 2016; De Schuyter et al. 2024).

Pollen needed for plant reproduction can be transferred between individuals by different pollinating agents. Completely pollinator‐independent plant species use, for example, abiotic pollination by wind, selfing, or vegetative reproduction instead of pollinators. In contrast, strictly pollinator‐dependent species rely exclusively on pollen delivered by pollinators. If plants do not receive enough high‐quality pollen (Ashman et al. 2004; Knight et al. 2005), their fecundity and, eventually, plant population size may be reduced (Wilcock and Neiland 2002; Ashman et al. 2004; Rodger et al. 2021). High dependence on pollinators can make a species more susceptible to pollinator decline. However, complete reliance on pollinators rarely occurs in plant species, as most species can produce seeds without insect pollination (Goodwillie et al. 2005). The importance of pollinators varies across ecosystems, depending on the species composition within plant communities, shaped by a range of reproductive strategies.

Most plants reward pollinators with pollen and nectar (Potts et al. 2003; Willmer 2011). Therefore, the loss of pollen and nectar‐rewarding plants limits the amount of food available for pollinators (Potts et al. 2003; Blüthgen and Klein 2011; Burkle et al. 2013). Plant communities that provide enough food for pollinators should buffer against pollinator loss and subsequent negative effects on the plant reproduction of pollinator‐dependent plants (e.g., Nielsen et al. 2012; Lundgren et al. 2016). Pollinator‐dependent plant species may benefit from living in communities with high cover and a number of pollen‐ and nectar‐rewarding plants (Ghazoul 2006; Braun and Lortie 2019; Gavini et al. 2021; Alison et al. 2022, but see Johnson et al. 2022), especially with facilitating effects from the most visited (Biella et al. 2019) or the most nectar‐rich plant species that attract pollinators to the community (Carvalheiro et al. 2014).

Long‐term and large‐scale evidence on pollinator dependence and pollen‐nectar production of vascular plant communities is particularly limited in high‐latitude ecosystems. Importantly, high‐latitude ecosystems are strongly responding to changes in climate and land use (Post et al. 2009; Myers‐Smith et al. 2011). Tundra and boreal forests are the dominant ecosystems at high latitudes. In the tundra, observed changes include an increase in shrub cover and biomass (Myers‐Smith et al. 2011; Mekonnen et al. 2021), changes in plant structure and functions (Bjorkman et al. 2018), and shifting species dominance patterns (Bråthen et al. 2024). In boreal forests, temporal changes in plant communities have primarily been linked to land use, such as grazing (Happonen et al. 2021), forest management (Tonteri et al. 2016; Muurinen et al. 2019), and tourism and recreation activities (Tolvanen and Kangas 2016; Maliniemi and Virtanen 2021). As reviewed by Hederström et al. (2024), increased human land use may harm the reproduction of pollinator‐dependent plants more strongly than plants with pollinator‐independent strategies (Aguilar et al. 2006; Clough et al. 2014; Bennett et al. 2020) as it decreases pollinator abundance (Winfree et al. 2009; Gómez‐Martínez et al. 2020) and disrupts plant–pollinator interactions (Cirtwill et al. 2018).

The aim of this study is to provide ecosystem‐level evidence on how long‐term changes in vascular plant communities are reflected in the cover (%) of plant species that do or do not interact with pollinators. Such long‐term and large‐scale explorations are currently missing from the study area and are also relatively rare elsewhere (but see De Schuyter et al. 2024; Pan et al. 2024). We applied species‐specific ecological indicator values (EIVs; Tyler et al. 2021) to determine the average cover of pollinator‐dependent and pollinator‐independent plants, as well as pollen‐nectar and non‐nectar plants. The cover of pollinator‐dependent plants indicates the demand of plant communities for insect pollination, whereas the cover of pollen‐nectar plants indicates the provision of food for pollinators in terms of the availability of potential pollen‐nectar sources. Using spatially and temporally extensive vegetation plot resurvey data, we analyze changes in the cover of pollinator‐dependent and pollen and nectar‐rewarding plants between the 1960s and 2010s in two distinct ecosystems of northern Fennoscandia: tundra and boreal herb‐rich forests, and in both undisturbed and disturbed sites in terms of land use. The two ecosystems represent different ends of the productivity gradient in the study area. The tundra is a low‐productive system with slow process rates, whereas the herb‐rich forest represents a more productive system with faster process rates (Jonasson et al. 2000; Maliniemi et al. 2019). Specifically, we first quantify the average cover of vascular plant species in all different classes (EIVs) of pollinator dependence and pollen‐nectar production. Second, we analyze temporal changes in the average cover of pollinator‐dependent and pollinator‐independent plants and pollen‐nectar and non‐nectar plants between the 1960s and 2010s. Third, we explore which plant growth forms contribute to the observed cover changes.

## 2 Materials and Methods

### 2.1 Study Area and Temporal Vegetation Data

**FIGURE 1:**
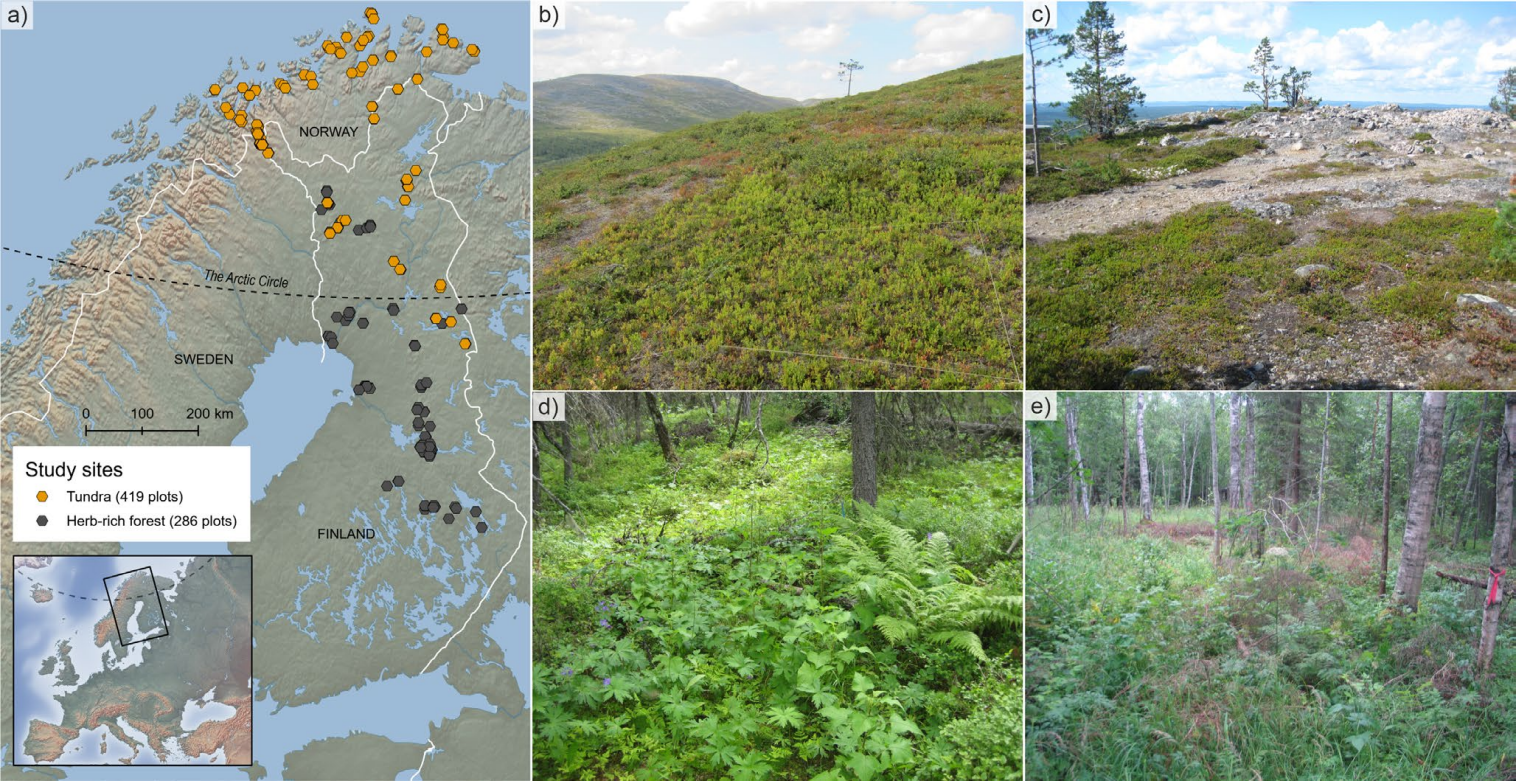
(a) Map of the study area and location of the study sites. Photos represent (b) undisturbed and (c) disturbed tundra plots and (d) undisturbed and (e) disturbed plots in herb‐rich forests. Map: Ala‐Hulkko, T. Background map and boundaries: Natural Earth (2024, naturalearthdata.com).

The study area is located in northern Fennoscandia (Figure 1a), covering latitudes 62.86°–71.12° N and longitudes 18.63°–30.73° E. This study includes treeless heath and oroarctic tundra vegetation (hereafter, tundra) and boreal herb‐rich forest vegetation. Tundra sites represent oligotrophic vegetation, dominated by dwarf shrubs ( *Empetrum nigrum* , *Vaccinium myrtillus* , and *Calluna vulgaris* ) or low shrubs ( *Betula nana* ) with often considerable cover of bryophytes and lichens. Herb‐rich boreal forests are a distinct, fertile forest site type, dominated by forbs (e.g., *Geranium sylvaticum* and *Filipendula ulmaria* ) and ferns (e.g., *Gymnocarpium dryopteris* and *Athyrium filix‐femina* ), and thus are biodiversity hotspots among the more species‐poor boreal forest types. Both ecosystems are of great conservation importance (Kontula and Raunio 2019). The pollinator species in the tundra are generally few and specialized. Main pollinators include *Bombus* species and species from the suborder *Brachycera*. In forests, main pollinators include, for example, *Bombus* species, wild bees, and moths and butterflies (Heliölä et al. 2021). However, there is very little information available in Finland about forests as pollinator habitats. Taxon nomenclature used here follows the FinBIF checklist of Finnish species (2024). The study area covers 13 different biogeographical regions. In Finland, regions mostly follow the borders of Finnish biogeographical provinces (Happonen et al. 2021), and in Norway, regions are delimited on the basis of boreal (coastal) and alpine (inland) zones and longitudinal gradient (i.e., county; Finnmark, Tromsø) (Bakkestuen et al. 2008). Information on biogeographical regions was used in the analyses to account for the geographical variation of environmental conditions, as the focus is on ecosystem‐level changes.

Tundra sites were originally sampled by Haapasaari (1988) during 1964–76 and herb‐rich forest sites by Kaakinen (1971, 1974, unpubl.) during 1968–75. Of these sites, we located and resampled a total of 419 tundra sites and 286 herb‐rich forest sites during 2013–2020 (described in detail in Maliniemi et al. 2018; Happonen et al. 2021; Bråthen et al. 2024). All sites were sampled once during the original survey and once during the resurvey, and temporal comparisons are made between these two timepoints. Resampling was carried out for all sites approximately at the same time of the growing season (i.e., comparable phenological state of the vegetation) as in the original survey (between June and August, depending on the geographical location). Original sites were located on the basis of detailed location information (e.g., description of specific place, elevation, slope, and aspect). Thus, study sites are quasi‐permanent (Kapfer et al. 2017), that is, their exact locations were not marked in the field during the original survey. The inherent relocation error was estimated to be generally small (Maliniemi et al. 2018; Happonen et al. 2021; Bråthen et al. 2024), and it was further minimized in the analyses by avoiding comparisons of changes in individual pairs of plots and instead focusing on comparing plot‐scale averages (medians) calculated for the 1960s and 2010s in both study environments. The identity and absolute cover (%) of each vascular plant species were visually estimated from 2 × 2 m plots in tundra sites and from 5 × 5 m plots in herb‐rich forest sites (including only understory species) using a percentage cover scale that was comparable between the original survey and resurvey. All analyses are based on absolute cover data.

Before analyses, we also divided the plots into undisturbed and disturbed sites on the basis of clear signs of recent land use disturbance visually observed during the resurvey. In the original survey, all sites were undisturbed by human land use. A total of 72 tundra sites and 143 herb‐rich forest sites had experienced clear land use disturbance by the resurvey, whereas 347 tundra sites and 143 herb‐rich forest sites had remained undisturbed. In herb‐rich forests, human disturbance was most often associated with forestry and forest management (Happonen et al. 2021), whereas in tundra, infrastructure and land use associated with ski resorts and tourism activities were the most common sources of human disturbance (Maliniemi et al. 2018; Bråthen et al. 2024). Thus, in this study, land use disturbance represents any general, major alteration of the study site. Notably, none of the study sites were affected by agricultural land use. The impact of semi‐domesticated reindeer grazing was also excluded from the land use, as its effect can be assumed similar across disturbance levels used (and its effects on vegetation changes have already been studied in Maliniemi et al. 2018 and Happonen et al. 2021).

### 2.2 Indicator Values for Pollinator Dependence and Pollen‐Nectar Production of Plant Species

We used EIVs from Tyler et al. (2021) on vascular plants of northern Europe to assess the EIVs of pollinator dependence and nectar production for each species in our data. In Tyler et al. (2021), discrete EIVs for pollinator dependence range from 0 (pollinator‐independent) to 2 (pollinator‐dependent) and for nectar production from 1 (no nectar or pollen production) to 7 (> 200 g of nectar produced/m^2^/year) (detailed EIVs in Table 1). In total, 132 species were recorded across the surveys in tundra and 228 species in herb‐rich forests (Tables S1, S2). If a species lacked an EIV for pollinator dependence or nectar production, we either inferred its EIV from closely related species, given that EIVs were similar within genera (the numbers of such species were 2 in tundra and 5 in herb‐rich forests), or removed species from the analyses (see removed species in Tables S1, S2). EIVs for pollinator dependence and nectar production were found for 124 and 129 species in tundra, and 219 and 224 species in herb‐rich forests.

### 2.3 Data Treatment and Analyses

To understand the overall variation and range within pollinator dependence and pollen–nectar production EIVs in the study environments, we calculated the average plot‐level cover of plants with all different EIVs for pollinator dependence and nectar production in the 1960s and 2010s in both tundra and herb‐rich forests. This was based on summing up the absolute cover of all species in a plot with similar pollinator dependence or nectar production EIV.

Next, to analyze temporal cover changes related to pollinator dependence and pollen‐nectar production of vascular plant communities, we separately examined the absolute cover of pollinator‐dependent, pollinator‐independent, pollen‐nectar, and non‐nectar plants as response variables, on the basis of the original EIVs. The strong variation found in plant covers within and among the original EIVs, and the logarithmic scale in measured nectar production EIV by Tyler et al. (2021) would have complicated the analyses of changes in the average nectar production when calculated as a (weighted or non‐weighted) mean of different EIV classes. We treated all species that relied to some degree on insect pollination as pollinator‐dependent plants. These included both self‐incompatible species considered strictly pollinator‐dependent (original EIV of 2) and species that use insect pollinators in addition to other pollinating agents (EIV 1; self‐compatible and wind‐ or water‐pollinated species). We included all species that produce at least some amount of nectar and/or pollen (species with original nectar production EIV 2–7) as pollen‐nectar plants, whereas species with no nectar or pollen production (EIV 1) were treated as non‐nectar plants. We calculated the absolute cover values of these groups by summing up the cover of pollinator‐dependent and pollinator‐independent and pollen‐nectar plants and non‐nectar plants in each vegetation plot. The absolute cover values inform about the decadal changes in plant species that require (pollinator‐dependent plants) and/or support pollinators (pollen‐nectar plants), and species not interacting with pollinators. Although this simplification averages out variation and masks the class‐specific EIV information (detailed in Table 1), our focus is on identifying major, ecosystem‐wide trends in vegetation that indicate shifts in ecosystem condition. Therefore, we prioritize documenting broad, significant shifts in pollinator dependence or pollen‐nectar availability over more specific but uncertain information (such as the amount of pollen and/or nectar produced per site per year).

Most species were both pollinator‐dependent and pollen‐nectar plants or pollinator‐independent and non‐nectar plants. However, both ecosystems included several species that were pollinator‐independent but produced pollen or nectar, whereas pollinator‐dependent species without pollen or nectar production were rare. To understand how pollinator dependence and nectar production are interrelated in the study environments, we calculated Spearman's correlations between pollinator‐dependent and pollen‐nectar species using the R package ‘BBcor’ (Williams and Rodriguez 2022). We used the response variables described above and included species that have value for both pollinator dependence and nectar production. All statistical analyses were conducted in R (version 4.3.3, R Core Team 2024).

For analyses, we used Bayesian linear regression models from R package ‘brms’ (version 2.22.0, Bürkner 2017), which utilizes the Bayesian modeling platform Stan (Carpenter et al. 2017) to analyze temporal changes in the response variables (pollinator‐dependent, pollinator‐independent, pollen‐nectar plants, and non‐nectar plants). We fitted separate models for each response variable using the Weibull distribution with a log‐link function. Weibull provides flexibility in modeling by varying distributional shapes of non‐negative, skewed cover responses through its shape (*k*) and scale (λ) parameters. The applicability of the Weibull distribution in ecology has been demonstrated, for example, in studies of species abundance distributions on the basis of relative species cover (Ulrich et al. 2018; Moradi et al. 2025). Compared to more commonly used distributions in the modeling of species (absolute) coverage, such as the log‐normal, the Weibull distribution offered several advantages for our data. It provided a better fit for the responses with varying degrees of skewness, including those exhibiting more symmetric distributional shapes. Additionally, its lighter‐tailed posterior distributions helped prevent unrealistically large predictions of maximum covers.

In the models, we added time (original vs. resurvey) and land use (undisturbed vs. disturbed) and their interaction as fixed effects. Hence, for all response variables in the analyses, each site included two observations: cover in the original survey and cover in the resurvey. We added plot identity as a random effect to account for repeated measurements and biogeographical region to account for potential geographical variation. We gave time and human disturbance a weakly informative normal (0, 50; where 0 = mean and 50 = standard deviation) prior that allowed for posterior predictions to show large but plausible changes in plant cover through time. More informative normal priors (e.g., with standard deviation closer to one) smoothed posterior predictions excessively between both time periods and disturbance levels when compared to the raw data. For group‐level (i.e., random) effects (plot identity and biogeographical region) and intercept, we gave ‘brms’ default half student‐*t* priors with three degrees of freedom and a scale parameter that differed depending on the standard deviation of the response (Bürkner 2017). In the intercept prior, the mean of the intercept was centered around the zero of the population‐level effects. We fitted all models with four Markov Chain Monte Carlo (MCMC) chains of 15,000 iterations, discarded the first 5000 samples of each chain as warmup, which then left 40,000 MCMC samples of each parameter of each model to ensure model convergence. To improve sampling efficiency and avoid divergent transitions during MCMC sampling, we increased the ‘adapt_delta’ parameter to 0.99 in all models. We estimated the average marginal effect of time on plant cover using the R library ‘emmeans’ (version 1.10.0, Lenth 2024) to quantify the average (median) change in cover over time. The results of all the models were also visualized by plotting the modeled average (median) cover of growth forms in the 1960s and 2010s separately for undisturbed and disturbed sites in both tundra and herb‐rich forests.

To further explore the temporal patterns, we analyzed temporal changes in the absolute cover of plant growth forms to understand which growth forms contributed to the observed changes in pollinator‐dependent, pollinator‐independent, pollen‐nectar, and non‐nectar plants. First, we assigned all species either as deciduous shrubs, evergreen shrubs, forbs, graminoids, or pteridophytes, and combined this information with pollinator dependence and nectar production EIVs, resulting in 16 different variables (e.g., pollinator‐dependent deciduous shrubs) in both study environments. The covers of the 16 variables were used as response variables in 'brms' models with Weibull distributions. Separate models were fitted for each variable, except for graminoid and pteridophyte species, which were exclusively pollinator‐independent non‐nectar plants; therefore, a single model was sufficient for these groups. We fitted the models with only time as a fixed effect with weakly informative normal (0, 50) priors, whereas plot identity and biogeographical region were added as random effects with 'brms' default priors. Additional models (Figure S1) were fitted with the interaction of time and land use added as fixed effects with normal (0, 50) priors, and plot identity and biogeographical region as random effects with 'brms' default priors.

## 3 Results

### 3.1 Average Plant Cover Across Detailed EIVs

**FIGURE 2:**
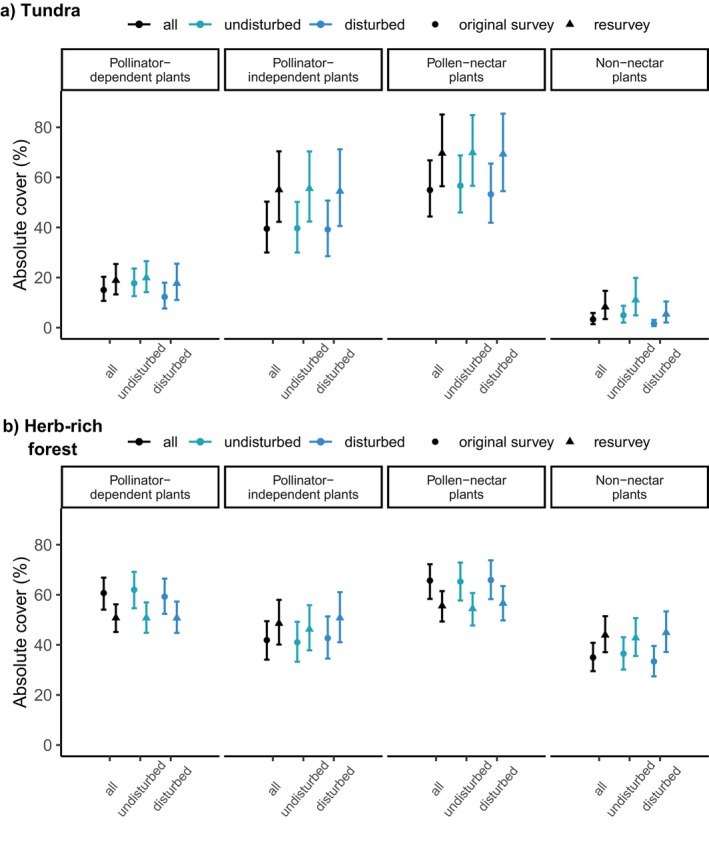
The average absolute cover of pollinator‐dependent and pollinator‐independent plants, and pollen‐nectar and non‐nectar plants across all sites and in undisturbed and disturbed sites in (a) tundra and (b) herb‐rich forests in the 1960s (original survey) and 2010s (resurvey). The estimates are modeled medians with 95% credible intervals.

When examining the detailed EIV range, the vascular plant cover in the tundra consisted mainly of pollinator‐independent species during both the 1960s (original survey) and 2010s (resurvey), whereas pollinator‐dependent species (EIVs 1–2) covered clearly less in both surveys (Table 1, Figure 2a). For most of the latter, pollination was facilitated by insects (EIV 1), whereas complete dependence on insect pollination (EIV 2) remained rare. Tundra sites' vascular plant cover was mostly composed of pollen‐nectar plants (Figure 2a), with species producing small amounts of nectar (0.2–5 g m^2^/year) but copious amounts of harvestable pollen (EIV 3) being most abundant in both surveys (Table 1).

**TABLE 1:** The absolute mean covers (%) ± SE of species belonging to different EIV classes across all plots in tundra and herb‐rich forests during both surveys. EIVs of pollinator dependence are based on Tyler et al. (2021) and are as follows: 0 = independent of pollinators, 1 = pollination facilitated by insects, in addition to selfing and/or pollination by abiotic agents, and 2 = exclusively pollinated by insects. Nectar production EIVs (Tyler et al. 2021) are as follows: 1 = no nectar production (0 g sugar/m^2^/year) and no collectable pollen, 2 = nectar production insignificant (< 0.2 g) or absent but with low but significant amounts of collectable pollen, 3 = small (0.2–5 g) or lower but with copious collectable pollen, 4 = nectar production modest (5–20 g), 5 = rather large (20–50 g), 6 = large (5–200 g), and 7 = very large (>200 g). N/A refers to species for which no EIV was found.

	Pollinator dependence EIV				Nectar production EIV							
	0	1	2	N/A	1	2	3	4	5	6	7	N/A
Tundra, 1960s	41.9 ± 1.4	19.1 ± 1.1	3.6 ± 0.6	1.0 ± 0.2	7.0 ± 0.6	0.2 ± 0.1	36.4 ± 1.4	5.2 ± 0.4	16.5 ± 1.1	0.3 ± 0.1	0.1 ± 0.1	0.1 ± 0.1
Tundra, 2010s	61.5 ± 1.7	20.6 ± 1.2	5.2 ± 0.6	1.2 ± 0.2	12.6 ± 0.8	0.2 ± 0.1	51.0 ± 1.7	9.4 ± 0.7	14.3 ± 0.9	0.5 ± 0.1	0.2 ± 0.1	0.1 ± 0.1
Forest, 1960s	46.5 ± 1.6	35.3 ± 1.3	26.7 ± 1.2	1.4 ± 0.1	38.9 ± 1.5	2.7 ± 0.2	43.9 ± 1.3	12.1 ± 0.8	8.4 ± 0.5	0.7 ± 0.1	1.2 ± 0.1	2.2 ± 0.2
Forest, 2010s	53.7 ± 1.8	33.2 ± 1.3	17.1 ± 0.9	0.8 ± 0.1	48.1 ± 1.7	2.3 ± 0.2	27.1 ± 1.1	11.7 ± 0.7	13.0 ± 0.9	0.5 ± 0.1	1.2 ± 0.1	0.9 ± 0.1

In herb‐rich forests, pollinator‐dependent species covered more than 50% on average during both surveys (Table 1, Figure 2b). Species with insect‐facilitated pollination (EIV 1) covered more during both surveys than species pollinated exclusively by insects (EIV 2). In terms of nectar production, a relatively high cover consisted of species that produce neither nectar nor pollen (EIV 1). Of the pollen‐nectar species, those producing 0.2–5 g (EIV 3) covered most, followed by EIVs 4 (5–20 g) and 5 (20–50 g). Spearman's correlation between pollinator dependence and nectar production (i.e., the correlation of species EIVs) was 0.75 (CI's 0.65, 0.85) in the tundra and 0.78 (0.70, 0.86) in herb‐rich forests.

### 3.2 Temporal Changes in the Cover of Pollinator‐Dependent, Pollinator‐Independent, Pollen‐Nectar, and Non‐Nectar Plants

**FIGURE 3:**
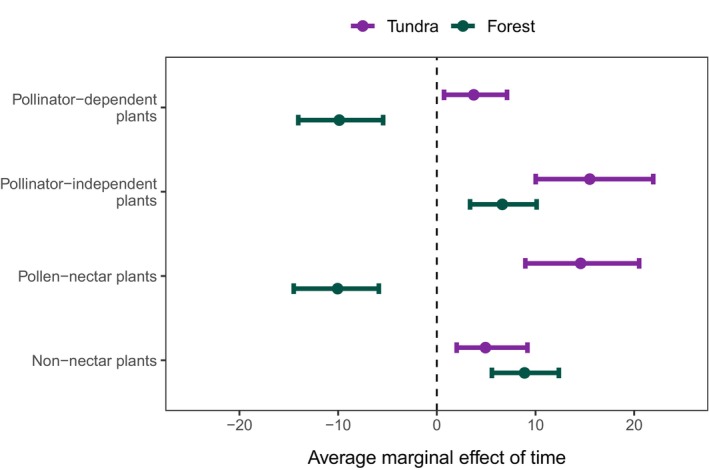
The estimated average marginal effect of time on the cover of pollinator‐dependent and pollinator‐independent plants and pollen‐nectar and non‐nectar plants in the tundra and herb‐rich forests. The estimates are modeled medians with 95% credible intervals.

Land use disturbance had little to no effect on the cover changes of all response variables (Figure 2, Figure S2). The modeling results showed an average increase of 15.5% (with 95% credible interval: 10.0 to 21.9) in the absolute cover of pollinator‐independent plants in the tundra from the 1960s to the 2010s (Figure 3). The average cover of pollinator‐dependent plants increased by 3.7% (CI: 0.7–7.1). The absolute cover of pollen‐nectar plants also increased strongly (14.6%, CI: 8.1–16.7), whereas the cover of non‐nectar plants increased by 4.9% (CI: 8.1–16.7 Figure 3).

In herb‐rich forests, the absolute cover of pollinator‐dependent plants decreased around 10% (CI: −14.0 to −5.4), along with a highly similar decrease in the cover of pollen‐nectar plants (Figure 3). Pollinator‐independent plants showed a minor cover increase of 6.6% (CI: 3.37–10.1; Figure 3), whereas the cover of non‐nectar plants increased especially in disturbed sites (11.5%, CI: 6.73–16.3; Figure S2). Although there were geographical differences in the estimated absolute covers, particularly for pollen‐nectar and pollinator‐independent plants in the tundra, the temporal trends remained consistent in magnitude and direction throughout the study area (Figure S3).

### 3.3 Contribution of Plant Growth Forms on the Observed Changes

**FIGURE 4:**
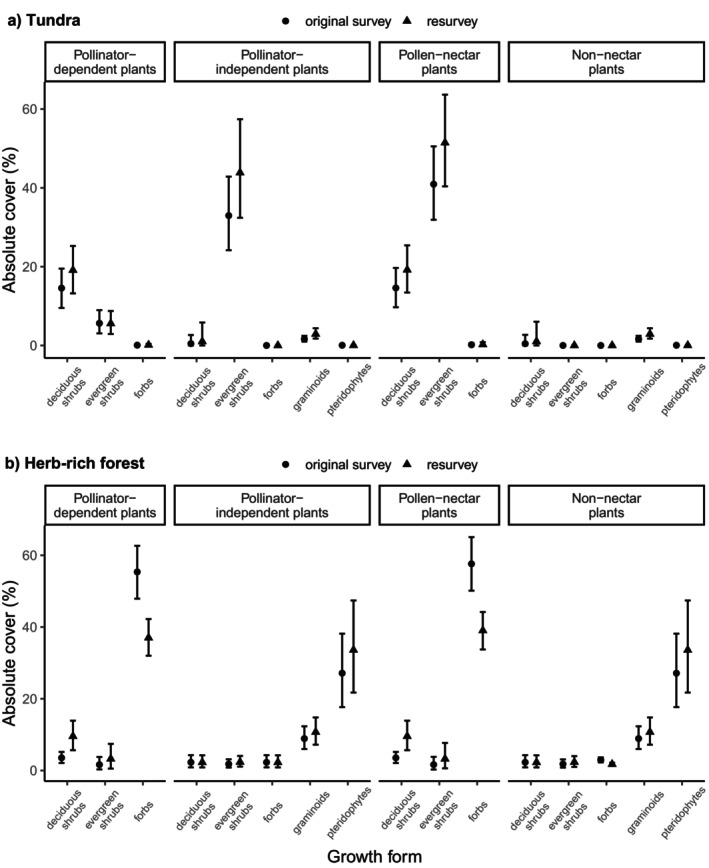
The contribution of different plant growth forms to changes in pollinator‐dependent and pollinator‐independent plants and pollen‐nectar and non‐nectar plants in the (a) tundra and (b) herb‐rich forests. The estimates are modeled medians with 95% credible intervals calculated across all sites.

The observed increase in the absolute cover of both pollinator‐independent and pollen‐nectar plants across the tundra sites (Figures 2a, 3) resulted from an increase in the cover of evergreen shrubs (Figure 4a; Figure S4). Model results also suggest that the minor increases observed in the cover of pollinator‐dependent and pollen‐nectar plants (Figures 2a, 3) were linked to increased deciduous shrub cover (Figure 4a; Figure S4).

In herb‐rich forests, the observed decrease in the absolute cover of pollinator‐dependent and nectar plants (Figures 2b, 3) resulted from a clear decrease in forb cover (Figure 4b; Figure S4). However, the increasing cover of pollinator‐dependent and pollen‐nectar deciduous shrubs counteracted the effect of the decrease in forb cover to some extent (Figure S4). The results of the additional models (see Figure S1) suggest that the increase in pteridophytes, which are all pollinator‐independent species that produce no pollen or nectar, occurred mainly in disturbed sites. This increase was mostly responsible for the rise in pollinator‐independent and non‐nectar plant cover.

## 4 Discussion

We explored whether changes in the composition of plant communities over time were reflected in shifts toward or away from pollinator dependence, as well as toward the increased availability or the absence of floral rewards in tundra and forest ecosystems. This study is among the first to quantify long‐term coverage changes that indicate shifts in insect pollination need and support by high‐latitude plant communities. In the tundra, pollinator‐independent plants consistently formed higher cover than pollinator‐dependent plants, with this disparity increasing over time. Similarly, pollen‐nectar plants exhibited greater cover than non‐nectar plants, with further increases noted in the resurvey. These changes were primarily driven by a substantial rise in evergreen shrub cover. Conversely, in herb‐rich forests, pollinator‐dependent and pollen‐nectar plants had slightly higher cover than their counterparts in the 1960s. However, by the resurvey, their cover had markedly decreased because of a decline in forbs and an increase in pteridophytes. In addition, we found that the observed changes were comparable between sites with land use disturbance and sites without disturbance. These results highlight how broad, global change‐driven structural vegetation changes can translate into regional‐scale shifts in ecosystem functioning. This informs future assessments of the condition of ecosystems undergoing rapid climate change.

### 4.1 Indication of Shifts in the Distribution of EIVs

Before grouping plant species into pollinator‐dependent or pollinator‐independent and pollen‐nectar or non‐nectar plants, we examined the more specific EIV range of these variables. The higher cover of pollinator‐independent than pollinator‐dependent plants in the tundra (Table 1) confirms the longstanding assumption that tundra plant communities rely fairly little on pollinators to reproduce, despite some species clearly depending on pollinators (see also, Kevan 1972). This is linked to a cold, nutrient‐poor environment and short flowering season, which limit the growth, flowering, and seedling establishment of pollinator‐dependent species (e.g., Klady et al. 2011; Moulton and Gough 2011) and pollinator visits (e.g., Vassvik et al. 2024). In addition, most pollinator‐dependent tundra plants seem to use other forms of reproduction besides insect pollination (see, cover of pollinator dependence EIV 1 in Table 1). For species that cannot reproduce vegetatively, being able to switch between selfing and insect pollination is extremely important in variable tundra conditions (Kalisz et al. 2004). Low pollinator dependence and the very low cover of exclusively pollinator‐dependent plants (EIV 2 in Table 1) further suggest that tundra plant communities may not be highly vulnerable to the decline of pollinators observed in several ecosystems across the globe (e.g., Burkle et al. 2013). However, as shown by Klady et al. (2011), increasing growing season temperatures may enhance the sexual reproduction of tundra plants, especially shrubs, increasing the need for pollinators.

More than half of the species coverage in the tundra consisted of species with low nectar but high pollen production (< 5 g/m^2^/year, nectar production EIV 3 in Table 1). However, species that produce a fair amount of nectar (5–50 g, EIVs 4–5) covered about 25% of the sites, which is a surprisingly high amount of nectar‐rewarding plants given the relatively low primary production. Although pollinator‐dependent plants mostly provide pollen and nectar (R_Spearman_ = 0.75), the proportion of nectar plant cover is much higher by comparison (Table 1). Since most of the vascular plant cover was formed by pollen‐nectar plants, pollinators should find plenty of food opportunities in the Fennoscandian tundra, but perhaps mostly in the form of pollen. The high cover of nectar‐rewarding plants in tundra is comparable to findings from dwarf shrub heaths in Britain that were found to be nationally significant habitats for nectar production (Baude et al. 2016). Likewise, high pollen production of tundra plant communities supports the previously found increase of pollen production towards the north (Cunha et al. 2022). High pollen production can act as an insurance against unpredictable pollination for pollinator‐dependent plants or relate to selection toward wind‐pollinated species with high pollen production (Cunha et al. 2022), such as *E. nigrum* in our study area. Unfortunately, it was not possible to estimate from the EIV data whether the species providing higher amounts of nectar (EIV 4–7) also produced lots of pollen.

In the herb‐rich forests, around half of the vascular plant cover in the resurvey consisted of species that depend on pollinators to some extent, yet complete dependence on pollinators was fairly rare (Table 1). Similarly, half of the species do not produce any pollen or nectar despite the fairly productive environment. The high share of both pollinator‐independent plants and non‐nectar plants relates to the high cover of pteridophytes (e.g., ferns) and graminoids (Figure 4b, Table S2). As evidenced by Baude et al. (2016) in Britain, productive forests may produce almost as much nectar per habitat area as grasslands that are often regarded as the main pollinator habitats. Such forests have also been shown to harbor high floral cover and pollinator abundance (Alison et al. 2022). However, forests as pollinator habitats remain a relatively understudied topic (Ulyshen et al. 2023, but see Gómez‐Martínez et al. 2020) despite their potential to produce large amounts of nectar across Europe (De Schuyter et al. 2024).

### 4.2 Varying Temporal Responses but Little Effect of Land Use on Any Modeled Cover Variables

Although the average cover of pollinator‐dependent plants increased slightly in the tundra, the stronger increase in the cover of pollinator‐independent plants (see Figures 2a, 3) suggests that plant communities depend even less on insect pollination than they did 50 years ago. Though increasing pollinator independence aligns with trends observed elsewhere (Ehlers et al. 2021; Pan et al. 2024), this was not driven by the replacement of pollinator‐dependent plants. Although further research is needed to understand the effects of increasing relative pollinator independence on plant–pollinator interactions, these findings provide an interesting perspective on the geographical pattern of pollinator dependence (see, e.g., Ollerton et al. 2011; Moeller et al. 2017; Cunha et al. 2022). Conversely, these communities seem to now provide more pollen‐nectar sources for pollinators to feed on (Figures 2a, 3). However, this increase relates particularly to species that produce plenty of pollen but only small amounts of nectar (nectar production EIV 3, Table 1), especially *E. nigrum* . Increased cover of pollen‐nectar plants is somewhat in line with Baude et al. (2016) who found increased nectar production in British dwarf shrub heaths between 1978 and 2007. Taken together, the expansion of pollinator‐independent plants and pollen‐nectar plants that produce little nectar may increase competition between pollinator‐dependent plants (Braun and Lortie 2019; Johnson et al. 2022), but the increased availability of food plants for pollinators may also facilitate pollinator‐dependent plants (Ghazoul 2006; Braun and Lortie 2019; Gavini et al. 2021). Nevertheless, further research is necessary to understand the impact of this shift on the production of pollen and nectar, and on plant–pollinator interactions.

In herb‐rich forests, the strong decline in the average cover of both pollinator‐dependent and pollen‐nectar plants, along with the concurrent increase in pollinator‐independent plants that do not provide pollen or nectar (Figures 2b, 3), suggests that herb‐rich forest communities both require and support insect pollination less than before. We found a high correlation between pollinator‐dependent and pollen‐nectar species, but unlike in the tundra, the temporal responses of pollinator‐dependent plants and pollen‐nectar plants in forests were very similar. The decline of pollinator dependence corresponds to the observations of Hedwall and Brunet (2016) in boreal and temperate forests of Sweden. The apparent stronger decline in the cover of strictly pollinator‐dependent plants (EIV 2 in Table 1; change not modeled) than in plants using alternative pollinating agents in addition to pollinators (EIV 1) prompts the question of whether the self‐incompatible species are more susceptible to ongoing changes in herb‐rich forests (Aguilar et al. 2006; Clough et al. 2014; Bennett et al. 2020; Ehlers et al. 2021).

The decreased cover of pollen‐nectar plants likely has implications for nectar production in herb‐rich forests. The suggested small increase in pollen‐nectar plants with rather large nectar production (5–200 g/m^2^/year, EIV 5 in Table 1) may help offset the pronounced decrease of pollen‐nectar plants that provide only small amounts of nectar (EIV 3). Thus, total nectar levels may have remained stable in the herb‐rich forests despite the observed general decline in the cover of pollen‐nectar plants (Figures 2b, 3), contrary to the findings of De Schuyter et al. (2024) who found a long‐term decrease in potential nectar production in temperate forest herb layers. Potential increases in nectar‐rich species can also help the pollination of nectar‐poor species (Carvalheiro et al. 2014). However, the general decline in pollen‐nectar plants indicates that pollinators are, on average, less likely to find food sources from herb‐rich forests (Blüthgen and Klein 2011; Gómez‐Martínez et al. 2020).

The observed temporal trends seem largely independent of land use disturbance despite previous evidence of the effect of land use on plant species trait composition or diversity in the studied ecosystems (Happonen et al. 2021; Maliniemi and Virtanen 2021). Whereas land use can directly harm pollinator‐dependent plants more (Aguilar et al. 2006; Clough et al. 2014), their decline in forests was likely related to other factors that uniformly decreased their cover while favoring many pollinator‐independent species. Yet, the land use in this study is a mixture of different types and intensities, representing moderate, spatially variable land use on average, rather than extreme habitat loss and fragmentation that might restructure plant community composition (Hederström et al. 2024) and pollinators more strongly over time (Winfree et al. 2009). Indeed, even though our results show no or little effects of land use disturbance on the observed changes, the result could be different if land use consisted only of a certain form of strong land use, such as clear cuttings in forests.

### 4.3 Changes in Modeled Cover Responses Are Linked to Dominant Plant Growth Forms

Changes in the cover of dominant plant growth forms clearly influenced the observed trends in the cover of pollinator‐dependent, pollinator–independent, pollen‐nectar, and non‐nectar plants in both environments (Figures 2, 3, 4; Figures S1a,b, S4). This highlights the role of dominant growth forms in driving the geographical patterns of pollinator dependence and plant mating systems (Moeller et al. 2017). The expansion of evergreen dwarf shrubs (Figure 4; Figure S4), particularly *E. nigrum*, which is pollinator‐independent but produces lots of pollen and some nectar (Table S1b), contributed strongly to the observed increase in both pollinator‐independent and pollen‐nectar plant covers in the tundra. Highly competent, wind‐pollinated *E. nigrum* mainly reproduces vegetatively, and its individuals may live for decades (Tybirk et al. 2000). Of the pollinator‐dependent tundra plants in our data, all but one provide nectar (Table S1a,d). Therefore, the minor increase found in pollinator‐dependent plants (Figures 2a, 3) suggests added pollen and nectar supply for pollinators even without the effect of *E. nigrum*, which was supported by an additional post hoc model (Figure S5).

The increase in non‐nectar plant cover in the tundra (Figures 2a, 3) was linked to the increased deciduous shrub cover (Figure 4a; Figure S4), especially *B. nana* (Table S1b). The increase of two dominant shrub species, *B. nana* and *E. nigrum* , shows that the changes in the cover of pollen‐ and nectar‐producing species in the tundra are dependent on the identity and changes in both dominant species and expanding shrubs. This is important, given the ongoing shrubification across the circumpolar tundra (Myers‐Smith et al. 2011; Mekonnen et al. 2021). However, the patterns observed in our dwarf shrub‐dominated study area may not hold across all tundra regions, particularly where shrubification is driven by other taxa, such as willows (*Salix* spp.), widely known for both their high pollinator dependence and nectar production (see also Table S1a). In addition to shifts in dwarf shrub dominance, shrubification and increased productivity were also reflected in increased total cover across all modeled variables (see Table 1, Figure 2a).

In herb‐rich forests, the decrease in the cover of pollinator‐dependent and pollen‐nectar plants was mainly related to the decrease in forb cover (Figure 4b; Figures S1b, S4), especially that of large herbs (Table S2a). In the study area, this has been linked to reindeer grazing, as they prefer large‐sized forbs and consequently reduce their size and cover over time (Happonen et al. 2021). In addition, increased shade because of canopy closure (both natural succession and forest management) can hamper the growth of shade‐intolerant forbs (Happonen et al. 2021) and result in reduced nectar production (De Schuyter et al. 2024). Importantly, such direct declines of pollinator food plants can explain the previously found negative effects of land use on pollinators (Roulston and Goodell 2011). A minor increase in the cover of pollinator‐dependent shrubs producing pollen and nectar (Figure 4b; Figure S4) did not alleviate the overall losses in the cover of pollinator‐dependent and pollen‐nectar plants (Figures 2b, 3). The decline of forbs is troubling, as they provide the most abundant (Figure 4b) and diverse (Table S2a,b) nectar source for pollinators in herb‐rich forests, and likely vary in traits that shape plant–pollinator interactions, such as flowering time, floral shapes, and colors (Ghazoul 2006; Blüthgen and Klein 2011). A strong decline in the two most abundant large herb species ( *G. sylvaticum* , *F. ulmaria* ) contributed to the decrease of species with small amounts of nectar but copious pollen (nectar production EIV 3 in Table 1), which may harm pollinators through decreased pollen provision. Such plant species with low nectar production are likely more threatened by pollinator loss than nectar‐rich ones, because of them being less attractive for pollinators (Braun and Lortie 2019).

### 4.4 Links to Ecosystem Condition Assessments, Potential Uncertainties, and Future Studies

Long‐term time series on pollination or pollinating insects are rare or lacking from high‐latitude environments (but see Høye et al. 2013; Zoller et al. 2023). Yet, changes in the cover of pollinator‐dependent and pollen‐nectar plants could be used as indicators that provide complementary perspectives when assessing pollination as part of ecosystem functioning and condition, for example, in the proposed EU Pollinator Monitoring Scheme (EU‐PoMS, Potts et al. 2024; see also Maes et al. 2018; Pedersen et al. 2021). Such knowledge has been previously largely missing for most regions in Europe (but see Ehlers et al. 2021; Pan et al. 2024).

The cover of pollinator‐dependent plants reflects the importance of pollinators for the ecosystem. Therefore, more efficient insect pollination is needed to maintain the ecosystem functions provided by plant communities with a high degree of pollinator dependence (Hederström et al. 2024). Still, although their cover changes may reflect changes in insect pollination (e.g., Lundgren et al. 2016), plant reproduction represents only one of the factors affecting plant population size and hence community composition. This is pronounced in tundra, where reproduction through seeds is limited and less likely to affect plant populations (but see, Klady et al. 2011). The extent to which the observed declines in pollinator‐dependent plants in the herb‐rich forests are related to pollination requires temporal evidence on pollinator communities and plant reproduction. This is because a consistent decline in both pollinator‐dependent plants and pollinators can happen because of similar reactions to habitat change (Moser et al. 2015). Intriguingly, exploring changes in reproductive traits in relation to vegetative traits (Hederström et al. 2024) could reveal patterns driving not only the changes in pollinator dependence of plant communities but also the subsequent changes in ecosystem functions.

The cover of pollen‐nectar plants, in turn, relates to the availability of pollen‐nectar sources for pollinators. Yet, pollen‐nectar plant cover represents a more general abundance of species that provide food for pollinators and does not equal, for example, the cover of flowers (e.g., Alison et al. 2022). For example, large‐leaved forbs, such as *G. sylvaticum* , have few flowers compared to their areal coverage. However, ecosystem‐wide cover of pollen‐nectar plants can be calculated from existing long‐term vegetation data to complement direct measurements of pollen and nectar production, which can vary even within species (Willmer 2011). In arctic‐alpine communities, where pollinators tend to forage on various plants (Elberling and Olesen 1999), the cover of pollen‐nectar plants should represent the availability of food for most pollinators. In such ecosystems with low flower cover, increasing pollen‐nectar plant cover may prove most beneficial for pollinators (Alison et al. 2022). Community turnover that led to the widespread loss of pollen‐nectar plants in herb‐rich forests can restructure plant–pollinator interactions (Cirtwill et al. 2018) and make plant communities more vulnerable to the effects of global change.

The cover of pollinator‐dependent and pollen‐nectar plants can reflect similar trends, though this relationship may not always hold true. In the tundra, a single species ( *E. nigrum* ) that was pollinator‐independent yet produced pollen and low amounts of nectar dominated the cover change. This finding emphasizes the importance of considering both pollinator dependence and pollen and nectar production in future studies, as a change in one does not necessarily reflect a change in the other. Although our study focused only on species cover, future research should also consider the diversity of pollinator‐dependent (e.g., Ehlers et al. 2021) and pollen‐nectar plants, as their temporal, spatial, and floral diversity supports pollinators throughout the flowering season and ensures effective pollination for most plants (Ghazoul 2006; Blüthgen and Klein 2011). Still, beyond aggregated community‐level changes, species‐ and family‐level shifts should not be overlooked, as they may have distinct implications for plant–pollinator interactions. For instance, the decline of forbs in herb‐rich forests may reflect phylogenetic patterns that could help explain more specific ecological consequences. A rigorous examination of the specific drivers of the observed vegetation changes and inclusion of plant–pollinator interactions would reveal the underlying causes behind the patterns found in herb‐rich forests, and guide management actions to halt the current trend.

## Author Contributions

**Petteri Kiilunen:** conceptualization (equal), data curation (lead), formal analysis (lead), visualization (lead), writing – original draft (lead), writing – review and editing (equal). **Tuija Maliniemi:** conceptualization (equal), formal analysis (supporting), investigation (equal), supervision (equal), visualization (supporting), writing – original draft (supporting), writing – review and editing (equal). **Janne Alahuhta:** conceptualization (equal), funding acquisition (equal), supervision (equal), writing – review and editing (equal). **John‐Arvid Grytnes:** investigation (equal), writing – review and editing (equal). **Risto Virtanen:** investigation (equal), writing – review and editing (equal). **Kari Anne Bråthen:** investigation (equal), writing – review and editing (equal). **Konsta Happonen:** formal analysis (supporting), investigation (equal), writing – review and editing (equal). **Jutta Kapfer:** investigation (equal), writing – review and editing (equal). **Lauralotta Muurinen:** investigation (equal), writing – review and editing (equal). **Maria W. Skalska‐Tuomi:** investigation (equal), writing – review and editing (equal). **Terhi Ala‐Hulkko:** conceptualization (equal), funding acquisition (supporting), supervision (equal), writing – review and editing (equal).

## Conflicts of Interest

The authors declare no conflicts of interest.

## Supporting information


Data S1:

## Data Availability

Data and code are available from Zenodo: https://doi.org/10.5281/zenodo.15600879 (Kiilunen et al. 2025).
