# Supplementary material for: Decades of Change in Vascular Plant Composition in High‐Latitude Ecosystems: Shifting Prevalence of Pollination Strategies
Source: Ecol Evol. 2025 Oct 15;15(10):e72288. doi: 10.1002/ece3.72288 (PMC12527644; doi:10.1002/ece3.72288)
Supplement: Supplementary file 1 — Data S1: [file ECE3-15-e72288-s001.docx]

Supporting Information for:

**Decades Of Change in Vascular Plant Composition in High-Latitude Ecosystems: Shifting Prevalence of Pollination Strategies** (2025)

Petteri Kiilunen, Tuija Maliniemi, Janne Alahuhta, John-Arvid Grytnes, Risto Virtanen, Kari Anne Bråthen, Konsta Happonen, Jutta Kapfer, Lauralotta Muurinen, Maria W. Skalska-Tuomi & Terhi Ala-Hulkko

**Table S1.** List of tundra species, ordered by mean cover across all sites and grouped by **a)** pollinator-dependent nectar plants, **b)** pollinator-independent non-nectar plants, **c)** pollinator-independent nectar plants, **d)** pollinator-dependent non-nectar plants and **e)** species that miss EIV classification for either pollinator dependence, nectar production or both. Tables show absolute mean percentage cover in the original survey, mean cover change over time, EIV class for pollinator dependence and nectar production (based on Tyler et al. 2021, doi: 10.1016/j.ecolind.2020.106923) and species group in parentheses (dec = deciduous shrub, eve = evergreen shrub, forb = forb, gra = graminoid, pter = pteridophyte).

| **(a) Pollinator-dependent nectar plants** | | | | |
| --- | --- | --- | --- | --- |
| Species | Old mean cover (%) | Mean cover change (%) | Pollinator class | Nectar class |
| *Vaccinium myrtillus* (dec) | 12.1 | -3.1 | 1 | 5 |
| *Vaccinium vitis-idaea* (eve) | 3.2 | 0.5 | 1 | 3 |
| *Calluna vulgaris* (eve) | 3.2 | 0.7 | 2 | 5 |
| *Vaccinium uliginosum* (dec) | 2.2 | 3.4 | 1 | 4 |
| *Cornus suecica* (forb) | 1.1 | 0.8 | 1 | 3 |
| *Arctostaphylos uva-ursi* (eve) | 0.4 | 0.3 | 1 | 4 |
| *Linnaea borealis* (eve) | 0.2 | -0.1 | 1 | 2 |
| *Salix herbacea* (dec) | 0.2 | 0.1 | 2 | 6 |
| *Rubus chamaemorus* (forb) | 0.1 | 0.2 | 2 | 5 |
| **Species with old mean cover and mean cover change ≤ 0.1:** | | |  |  |
| *Achillea millefolium* (forb, Pollinator EIV 2, Nectar EIV 6)*; Andromeda polifolia* (eve, 1, 4); *Antennaria dioica* (dec, 2, 4); *Astragalus alpinus* (forb, 2, 5); *A. frigidus* (forb, 1, 5); *Bartsia alpina* (forb, 1, 5); *Campanula rotundifolia* (forb, 2, 4); *Chamaenerion angustifolium* (forb, 2, 6); *Dactylorhiza maculata* (forb, 2, 4); *Dryas octopetala* (forb, 2, 3); *Geranium sylvaticum* (forb, 2, 3); *Gymnadenia conopsea* (forb, 1, 4); *Lotus corniculatus* (forb, 2, 3); *Maianthemum biflora* (forb, 1, 3); *Melampyrum pratense* (forb, 1, 4); *M. sylvaticum* (forb, 1, 4); *Parnassia palustris* (forb, 2, 4); *Pinguicula alpina* (forb, 2, 4); *P. vulgaris* (forb, 1, 4); *Ranunculus acris* (forb, 2, 4); *Rhinanthus minor* (forb, 1, 4); *Rhodiola rosea* (forb, 1, 5); *Rhododendron tomentosum* (eve, 1, 4); *Rubus saxatilis* (forb, 2, 4); *Saussurea alpina* (dec, 2, 5); *Salix caprea* (dec, 2, 7); *S. glauca (dec, 2, 7); S. hastata* (dec, 2, 7); *S. lanata* (dec, 2, 7); *S. lapponum* (dec, 2, 7); *S. myrsinites* (dec, 2, 7); *S. phylicifolia* (dec, 2, 7); *S. polaris* (dec, 2, 6); *S. reticulata* (dec, 2, 6); *Scorzoneroides autumnalis* (forb, 1, 5); *Sibbaldia procumbens* (forb, 1, 3); *Silene acaulis* (forb, 1, 4); *Solidago virgaurea (forb, 1, 5); Sorbus aucuparia* (dec, 2, 4); *Trollius europaeus* (forb, 2, 3); *Vicia cracca* (forb, 2, 4); *Viola biflora* (forb, 1, 3) | | | | |

| **(b) Pollinator-independent non-nectar plants** | | | | |
| --- | --- | --- | --- | --- |
| Species | Old mean cover (%) | Mean cover change (%) | Pollinator class | Nectar class |
| *Betula nana* (dec) | 5.0 | 3.6 | 0 | 1 |
| *Avenella flexuosa* (gra) | 0.8 | 0.7 | 0 | 1 |
| *Carex bigelowii* (gra) | 0.2 | 0.2 | 0 | 1 |
| *Calamagrostis lapponica* (gra) | 0.2 | -0.1 | 0 | 1 |
| *Oreojuncus trifidus* (gra) | 0.2 | 0.1 | 0 | 1 |
| *Juniperus communis* (eve) | 0.1 | 0.3 | 0 | 1 |
| *Betula pubescens* (dec) | < 0.1 | 0.3 | 0 | 1 |
| **Species with old mean cover and mean cover change ≤ 0.1:** | | |  |  |
| *Agrostis capillaris* (gra); *A. mertensii* (gra); *Anthoxanthum nipponicum* (gra); *Athyrium distentifolium* (pter); *Calamagrostis neglecta* (gra); *C. phragmitoides* (gra); *Carex brunnescens* (gra); *C. canescens* (gra); *C. glacialis* (gra); *C. globularis* (gra); *C. lachenalii* (gra); *C. nigra* (gra); *C. pauciflora* (gra); *C. vaginata* (gra); *Deschampsia cespitosa* (gra); *Diphasiastrum alpinum* (pter); *D. complanatum* (pter); *Equisetum arvense* (pter); *E. palustre* (pter); *E. pratense* (pter); *E. sylvaticum* (pter); *Eriophorum angustifolium* (gra); *E. vaginatum* (gra); *Festuca ovina* (gra)*; F. rubra* (gra); *F. vivipara* (gra); *Gymnocarpium dryopteris* (pter); *Hierochloë alpina* (gra); *Huperzia arctica* (pter); *Juncus filiformis* (gra); *Luzula arcuata* (gra); *L. multiflora* (gra); *L. pilosa* (gra); *L. spicata* (gra); *L. sudetica* (gra); *Lycopodium clavatum* (pter); *Nardus stricta* (gra); *Phegopteris connectilis* (pter); *Picea abies* (eve); *Pinus sylvestris* (eve); *Plantago major* (forb); *Pyrola minor* (forb)*; P. rotundifolia* (forb); *Rumex acetosa* (forb); *R. acetosella* (gra); *Selaginella selaginoides* (pter); *Spinulum annotinum* (pter); *Trichophorum cespitosum* (gra) | | | | |

| **(c) Pollinator-independent nectar plants** | | | | |
| --- | --- | --- | --- | --- |
| Species | Old mean cover (%) | Mean cover change (%) | Pollinator class | Nectar class |
| *Empetrum hermaphroditum* (eve) | 32.1 | 13.3 | 0 | 3 |
| *Arctous alpina* (eve) | 1.6 | 0.1 | 0 | 4 |
| *Kalmia procumbens* (eve) | 0.9 | 0.2 | 0 | 4 |
| **Species with old mean cover and mean cover change ≤ 0.1:** | | |  |  |
| *Antennaria alpina* (forb, Pollinator EIV 0, Nectar EIV 4); *Bistorta vivipara* (forb, 0, 3); *Cerastium fontanum* (forb, 0, 3); *Drosera rotundifolia* (forb, 0, 2); *Erigeron uniflorus* (forb, 0, 4); *Euphrasia wettsteinii* (forb, 0, 3); *Gnaphalium supinum* (forb, 0, 2); *Hieracium sp.* (forb, 0, 4); *Lysimachia europaea* (forb, 0, 2); *Oxyria digyna* (forb, 0, 2); *Populus tremula* (dec, 0, 3); *Vaccinium oxycoccos* (eve, 0, 4); *Veronica alpina* (forb, 0, 3) | | | | |

| **(d) Pollinator-dependent non-nectar plants** | | | | |
| --- | --- | --- | --- | --- |
| Species | Old mean cover (%) | Mean cover change (%) | Pollinator class | Nectar class |
| *Orthilia secunda* (forb) | < 0.1 | 0.0 | 1 | 1 |

| **(e) Species with unknown EIV class** | | | | |
| --- | --- | --- | --- | --- |
| Species | Old mean cover (%) | Mean cover change (%) | Pollinator class | Nectar class |
| *Phyllodoce caerulea* (eve) | 0.9 | 0.0 | N/A | 5 |
| **Species with old mean cover and mean cover change ≤ 0.1:** | | |  |  |
| *Alchemilla alpina* (forb, Pollinator EIV n/a, Nectar EIV 3); *Cassiope tetragona* (eve, n/a, 4); *Diapensia lapponica* (eve, n/a, n/a); *Harrimanella hypnoides* (eve, n/a, 4); *Pedicularis lapponica* (forb, n/a, 5); *Poa* sp. (gra, n/a, n/a); *Thalictrum alpinum* (forb, n/a, 1); *Tofieldia pusilla* (forb, 1, n/a) | | | | |

**Table S2.** List of herb-rich forest species, ordered by total mean cover across all sites and grouped by **a)** pollinator-dependent nectar plants, **b)** pollinator-independent non-nectar plants, **c)** pollinator-independent nectar plants, **d)** pollinator-dependent non-nectar plants and **e)** species that miss EIV classification for either pollinator dependence, nectar production or both. Tables show absolute mean cover in the original survey, mean cover change over time, EIV class for pollinator dependence and nectar production (based on Tyler et al. 2021, doi: 10.1016/j.ecolind.2020.106923) and species group in parentheses (dec = deciduous shrub, eve = evergreen shrub, forb = forb, gra = graminoid, pter = pteridophyte).

| **(a) Pollinator-dependent nectar plants** | | | | |
| --- | --- | --- | --- | --- |
| Species | Old mean cover (%) | Mean cover change (%) | Pollinator class | Nectar class |
| *Geranium sylvaticum* (forb) | 17.2 | -9.6 | 2 | 3 |
| *Filipendula ulmaria* (forb) | 11.9 | -5.1 | 1 | 3 |
| *Rubus saxatilis* (forb) | 6.3 | -1.1 | 2 | 4 |
| *Maianthemum biflora* (forb) | 3.3 | -0.9 | 1 | 3 |
| *Lactuca alpina* (forb) | 1.8 | -0.7 | 1 | 4 |
| *Viola epipsila* (forb) | 1.7 | -0.4 | 1 | 4 |
| *Prunus padus* (dec) | 1.5 | 2.4 | 1 | 3 |
| *Crepis paludosa* (forb) | 1.5 | -0.7 | 1 | 5 |
| *Convallaria majalis* (forb) | 1.2 | 0.7 | 1 | 5 |
| *Rubus idaeus* (forb) | 1.2 | 1.8 | 1 | 4 |
| *Geum rivale* (forb) | 1.0 | -0.5 | 1 | 5 |
| *Cirsium helenioides* (forb) | 1.0 | -0.2 | 1 | 4 |
| *Solidago virgaurea* (forb) | 1.0 | -0.1 | 1 | 7 |
| *Ribes spicatum* (dec) | 0.7 | 0.2 | 1 | 5 |
| *Fragaria vesca* (forb) | 0.7 | -0.3 | 1 | 5 |
| *Angelica sylvestris* (forb) | 0.7 | -0.3 | 1 | 3 |
| *Melampyrum sylvaticum* (forb) | 0.6 | -0.3 | 1 | 4 |
| *Viola mirabilis* (forb) | 0.6 | -0.2 | 2 | 5 |
| *Vaccinium vitis-idaea* (eve) | 0.5 | 1.1 | 1 | 3 |
| *Sorbus aucuparia* (dec) | 0.5 | 1.2 | 1 | 3 |
| *Chamaenerion angustifolium* (forb) | 0.5 | 0.0 | 2 | 4 |
| *Anthriscus sylvestris* (forb) | 0.4 | 0.0 | 2 | 6 |
| *Vaccinium myrtillus* (dec) | 0.4 | 1.5 | 1 | 5 |
| *Actaea spicata* (forb) | 0.3 | 0.0 | 1 | 5 |
| *Linnaea borealis* (eve) | 0.3 | 0.3 | 1 | 2 |
| *Trollius europaeus* (forb) | 0.3 | -0.2 | 1 | 2 |
| *Viola selkirkii* (forb) | 0.3 | -0.1 | 2 | 3 |
| *Daphne mezereum* (eve) | 0.2 | -0.1 | 1 | 3 |
| *Ranunculus acris* (forb) | 0.2 | -0.1 | 1 | 5 |
| *Stellaria nemorum* (forb) | 0.2 | 0.3 | 2 | 4 |
| *Silene dioica* (forb) | < 0.1 | 0.4 | 2 | 3 |
| **Species with old mean cover and mean cover change ≤ 0.1:** | | |  |  |
| *Acer platanoides* (dec, Pollinator EIV 2, Nectar EIV 6); *Achillea millefolium* (forb, 2, 5); *Actaea erythrocarpa* (forb, 1, 3); *Anemone nemorosa* (forb, 1, 2); *Angelica archangeliga* (forb, 2, 6); *Antennaria dioica* (forb, 2, 4); *Barbarea stricta* (forb, 1, 4); *Caltha palustris* (forb, 1, 3); *Campanula cervicaria* (forb, 1, 4); *C. persicifolia* (forb, 2, 4); *C. rotundifolia* (forb, 2, 4); *Centaurea phrygia* (forb, 2, 6); *Circaea alpina* (forb, 1, 5); *Cirsium arvense* (forb, 2, 5); *C. palustre* (forb, 1, 7); *Cornus suecica* (forb, 1, 5); *Dactylorhiza maculata* (forb, 2, 4); *Epipactis atroruben*s (forb, 2, 4); *Epipogium aphyllum* (forb, 1, 3); *Galium album* (forb, 1, 2); *G. boreale* (forb, 1, 3); *G. palustre* (forb, 1, 2); *Glechoma hederacea* (forb, 2, 3); *Goodyera repens* (forb, 2, 4); *Hepatica nobilis* (forb, 1, 2); *Heracleum sphondylium* (forb, 1, 6); *Hypericum maculatum* (forb, 1, 2); *Lathyrus pratensis* (forb, 2, 5); *Leucanthemum vulgare* (forb, 1, 6); *Lonicera xylosteum* (dec, 1, 5); *Lysimachia thyrsiflora* (forb, 1, 3); *L. vulgaris* (forb, 1, 2); *Neottia ovata* (forb, 2, 4); *Parnassia palustris* (forb, 2, 4); *Pimpinella saxifraga* (forb, 2, 5); *Platanthera bifolia* (forb, 2, 4); *Polygonatum odoratum* (dec, 1, 4); *Potentilla erecta* (forb, 2, 3); *P. palustris* (forb, 2, 3); *Prunella vulgaris* (forb, 1, 5); *Ranunculus repens* (forb, 1, 2); *Rhamnus frangula* (dec, 1, 3); *Rhinanthus minor* (dec, 1, 4); *Ribes nigrum* (dec, 1, 3); *Rosa acicularis* (dec, 1, 3); *R. majalis* (dec, 2, 5); *Rubus chamaemorus* (forb, 2, 5); *Salix caprea* (dec, 2, 7); *S. glauca* (dec, 2, 7); *S. hastata* (dec, 2, 7); *S. lapponum* (dec, 2, 7); *S. myrsinifolia* (dec, 2, 7); *S. pentandra* (dec, 2, 7); *S. phylicifolia* (dec, 2, 5); *Saussurea alpina* (forb, 2, 6); *Scorzoneroides autumnalis* (forb, 1, 5); *Stachys sylvatica* (forb, 1, 4); *Stellaria graminea* (forb, 1, 3); *Trifolium pratense* (forb, 2, 6); *T. repens* (forb, 2, 5); *Tussilago farfara* (forb, 1, 3); *Vaccinium uliginosum* (dec, 1, 4); *Valeriana sambucifolia* (forb, 2, 7); *Veronica chamaedrys* (forb, 1, 3); *V. longifolia* (forb, 1, 5); *V. officinalis* (forb, 1, 4); *V. serpyllifolia* (forb, 1, 3); *Viburnum opulus* (dec, 1, 5); *Vicia cracca* (dec, 2, 4); *V. sepium* (forb, 1, 3); *Viola canina* (forb, 1, 2); *V. palustris* (forb, 1, 3); *V. riviniana* (forb, 1, 3) | | | | |

| **(b) Pollinator-independent non-nectar plants** | | | | |
| --- | --- | --- | --- | --- |
| Species | Old mean cover (%) | Mean cover change (%) | Pollinator class | Nectar class |
| *Gymnocarpium dryopteris* (pter) | 7.3 | -0.1 | 0 | 1 |
| *Athyrium filix-femina* (pter) | 5.8 | 0.6 | 0 | 1 |
| *Matteuccia struthiopteris* (pter) | 5.6 | -0.7 | 0 | 1 |
| *Melica nutans* (gra) | 2.8 | -0.8 | 0 | 1 |
| *Phegopteris connectilis* (pter) | 2.7 | 0.8 | 0 | 1 |
| *Dryopteris expansa* (pter) | 2.4 | 1.6 | 0 | 1 |
| *Milium effusum* (gra) | 1.6 | 0.2 | 0 | 1 |
| *Elymus caninus* (gra) | 1.2 | 0.5 | 0 | 1 |
| *Equisetum pratense* (pter) | 1.1 | 0.8 | 0 | 1 |
| *Juniperus communis* (eve) | 0.9 | -0.4 | 0 | 1 |
| *Equisetum sylvaticum* (pter) | 0.8 | 1.5 | 0 | 1 |
| *Diplazium sibiricum* (pter) | 0.7 | 0.2 | 0 | 1 |
| *Calamagrostis phragmitoides* (gra) | 0.6 | 1.2 | 0 | 1 |
| *Avenella flexuosa* (gra) | 0.5 | 0.3 | 0 | 1 |
| *Deschampsia cespitosa* (gra) | 0.5 | 0.1 | 0 | 1 |
| *Picea abies* (eve) | 0.5 | 0.8 | 0 | 1 |
| *Betula pubescens* (dec) | 0.4 | 1.6 | 0 | 1 |
| *Luzula pilosa* (gra) | 0.3 | -0.1 | 0 | 1 |
| *Carex vaginata* (gra) | 0.2 | -0.1 | 0 | 1 |
| *Carex digitata* (gra) | 0.2 | 0.1 | 0 | 1 |
| *Agrostis* sp. (gra) | 0.2 | -0.1 | 0 | 1 |
| *Pyrola rotundifolia* (forb) | 0.2 | -0.1 | 0 | 1 |
| *Pteridium aquilinum* (pter) | 0.2 | 0.0 | 0 | 1 |
| *Dryopteris carthusiana* (pter) | 0.2 | 0.5 | 0 | 1 |
| *Calamagrostis arundinacea* (gra) | 0.2 | 0.3 | 0 | 1 |
| *Pyrola minor* (forb) | 0.2 | 0.0 | 0 | 1 |
| *Poa pratensis* (gra) | < 0.1 | 0.2 | 0 | 1 |
| Urtica dioica (forb) | < 0.1 | 0.3 | 0 | 1 |
| *Calamagrostis canescens* (gra) | 0.0 | 0.2 | 0 | 1 |
| **Species with old mean cover and mean cover change ≤ 0.1:** | | |  |  |
| *Anthoxanthum odoratum* (gra); *Betula pendula* (dec); *Botrychium lanceolatum* (pter); *B. lunaria* (pter); *Botrypus virginianus* (pter); *Calamagrostis epigejos* (gra); *C. lapponica* (gra); *Carex brunnescens* (gra); *C. canescens* (gra); *C. cespitosa* (gra); *C. disperma* (gra); *C. echinata* (gra); *C. elongata* (gra); *C. flava* (gra); *C. globularis* (gra); *C. loliacea* (gra); *C. nigra* (gra); *C. norvegica* (gra); *C. ovalis* (gra); *C. pallescens* (gra); *C. rhynchophysa* (gra); *C. vesicaria* (gra); *Cinna latifolia* (gra); *Cystopteris fragilis* (pter); *C. montana* (pter); *Dactylis glomerata* (gra); *Dryopteris filix-mas* (pter); *Elymus repens* (gra); *Equisetum arvense* (pter); *E. fluviatile* (pter); *E. hyemale* (pter); *E. palustre* (pter); *E. scirpoides* (pter); *Eriophorum angustifolium* (gra); *Festuca ovina* (gra); *F. rubra* (gra); *Glyceria lithuanica* (gra); *Huperzia selago* (pter); *Juncus filiformis* (gra); *Larix sp.* (dec); *Luzula multiflora* (gra); *L. sudetica* (gra); *Lycopodium annotinum* (pter); *Molinia caerulea* (gra); *Phalaris arundinacea* (gra); *Phleum alpinum* (gra); *P. pratense* (gra); *Pinus sylvestris* (eve); *Plantago major* (forb); *Poa annua* (gra); *P. nemoralis* (gra); *P. trivialis* (gra); *Pyrola media* (forb); *Rumex acetosa* (forb); *R. acetosella* (forb); *Scirpus sylvaticus* (gra); *Selaginella selaginoides* (pter) | | | | |

| **(c) Pollinator-independent nectar plants** | | | | |
| --- | --- | --- | --- | --- |
| Species | Old mean cover (%) | Mean cover change (%) | Pollinator class | Nectar class |
| *Oxalis acetosella* (forb) | 5.1 | -2.2 | 0 | 3 |
| *Lysimachia europaea* (forb) | 1.6 | -0.8 | 0 | 2 |
| *Alnus incana* (dec) | 1.1 | 0.9 | 0 | 3 |
| *Populus tremula* (dec) | 0.2 | 0.0 | 0 | 3 |
| *Alchemilla* sp. (forb) | 0.2 | -0.1 | 0 | 3 |
| **Species with old mean cover and mean cover change ≤ 0.1:** | | |  |  |
| *Bistorta vivipara* (forb, Pollinator EIV 0, Nectar EIV 3); *Cerastium fontanum* (forb, 0, 3); *Empetrum nigrum* (eve, 0, 3); *Epilobium hornemannii* (forb, 0, 3); *E. montanum* (forb, 0, 3); *E. palustre* (forb, 0, 3); *Euphrasia frigida* (forb, 0, 3); *Galeopsis bifida* (forb, 0, 5); *Galium uliginosum* (forb, 0, 3); *Gnaphalium norvegicum* (forb, 0, 3); *Myosotis sylvatica* (forb, 0, 5); *Ranunculus auricomus* (forb, 0, 3); *Rorippa palustris* (forb, 0, 5); *Scutellaria galericulata* (forb, 0, 5); *Stellaria fennica* (forb, 0, 3); *S. longifolia* (forb, 0, 2); *S. media* (forb, 0, 3); *Thalictrum flavum* (forb, 0, 2) | | | | |

| **(d) Pollinator-dependent non-nectar plants** | | | | |
| --- | --- | --- | --- | --- |
| Species | Old mean cover (%) | Mean cover change (%) | Pollinator class | Nectar class |
| *Orthilia secunda* (forb) | 0.4 | -0.1 | 1 | 1 |
| **Species with old mean cover and mean cover change ≤ 0.1:** | | |  |  |
| *Calypso bulbosa* (forb, Pollinator EIV 2, Nectar EIV 1); *Coeloglossum viride* (forb, 1, 1); *Corallorhiza trifida* (forb, 2, 1); *Moneses uniflora* (forb, 1 ,1) | | | | |

| **(e) Species with unspecified EIVs** | | | | |
| --- | --- | --- | --- | --- |
| Species | Old mean cover (%) | Mean cover change (%) | Pollinator class | Nectar class |
| *Paris quadrifolia* (forb) | 1.9 | -1.3 | 1 | N/A |
| *Rubus arcticus* (forb) | 0.4 | -0.3 | N/A | 4 |
| *Galium triflorum* (forb) | 0.2 | -0.1 | N/A | 2 |
| **Species with old mean cover and mean cover change ≤ 0.1:** | | |  |  |
| *Coptidium lapponicum* (forb, Pollinator EIV n/a, Nectar EIV 2); *Galium trifidum* (forb, n/a, 2); *Neottia cordata* (forb, n/a, 4); *Poa alpigena* (gra, n/a, n/a); *Ranunculus cassubicus* (forb, n/a, n/a); *Stellaria borealis* (forb, n/a, 2); *Taraxacum sp.* (forb, n/a, n/a) | | | | |

**Figure S1**. Additional models for plant growth forms showing temporal changes in the absolute covers of all plant growth forms in relation to land use disturbance in a) tundra and b) herb-rich forest (see, *Materials and methods: Data treatment and analyses* for details on modelling framework with absolute cover models).


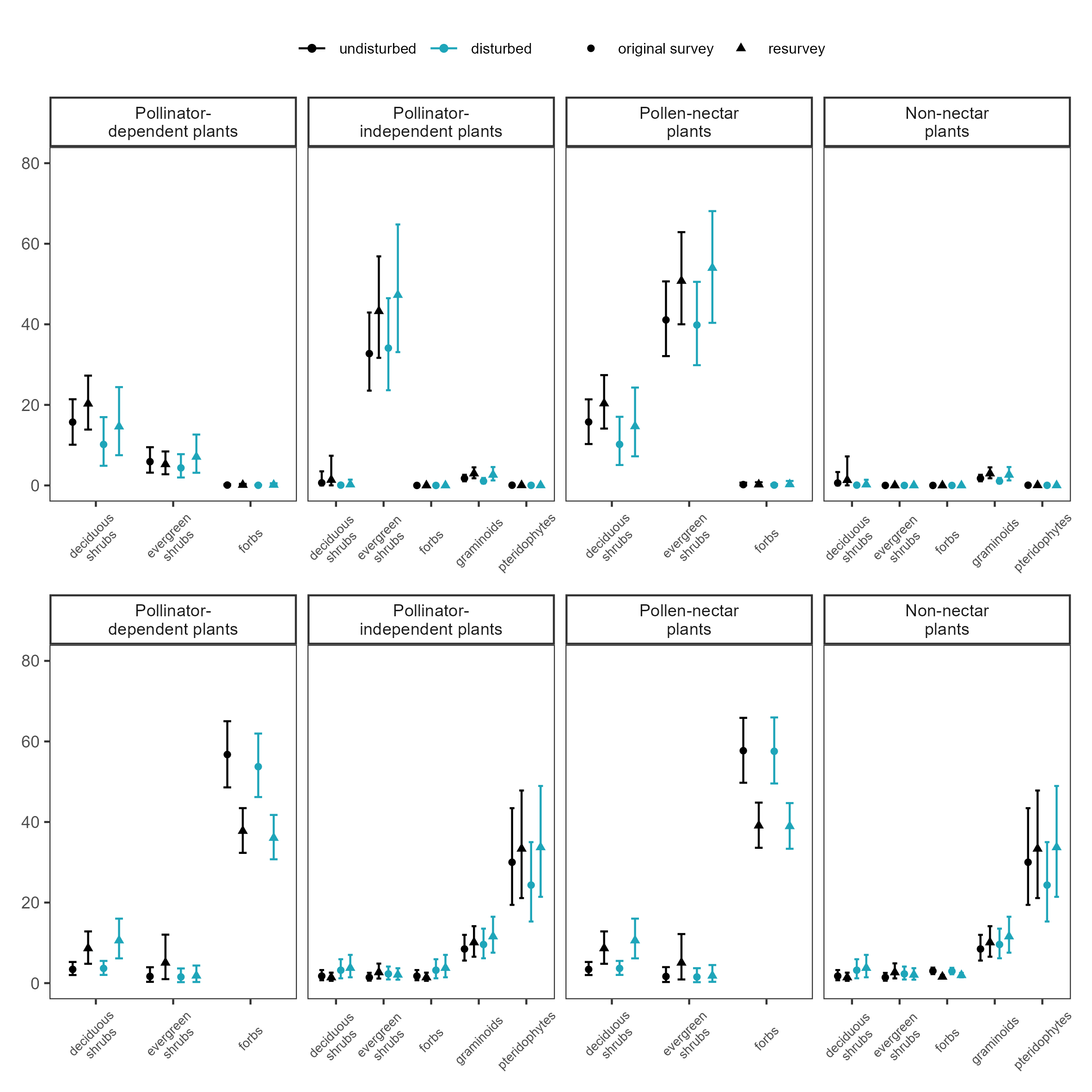


b)

a)


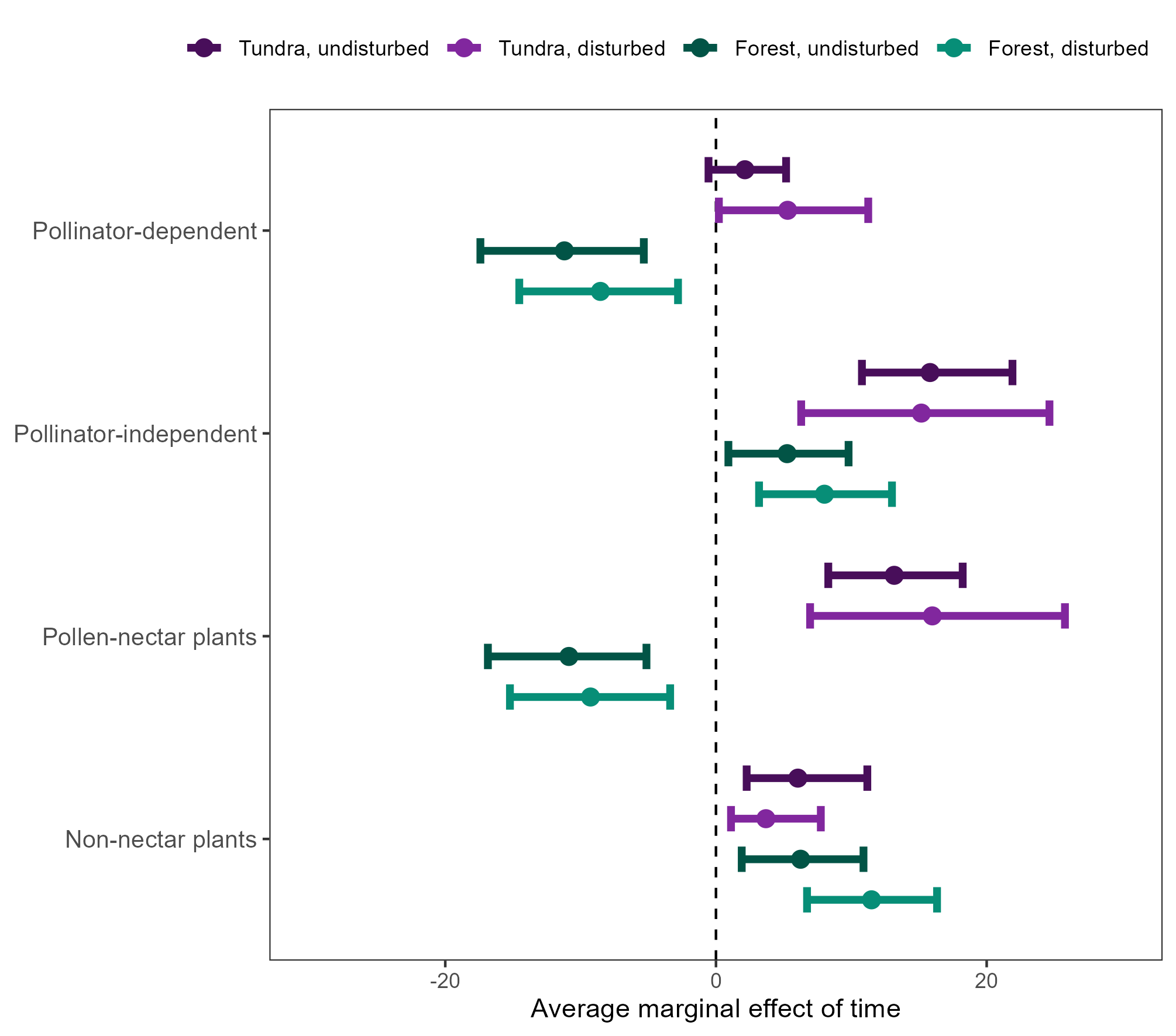


**Figure S2.** The estimated average marginal effect of time on the cover of pollinator-dependent and pollinator-independent plants and pollen-nectar and non-nectar plants conditioned for undisturbed and disturbed sites in the tundra and herb-rich forests. The estimates are modelled medians with 95% credible intervals.




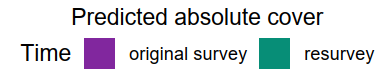


**Figure S3**. Posterior predictions of expected average absolute cover (%, x-axis) over the biogeographical regions (y-axis) in original survey and resurvey for a) pollinator-dependent, b) pollinator-independent, c) pollen-nectar and d) non-nectar plants in tundra and e) pollinator-dependent, f) pollinator-independent, g) pollen-nectar and h) non-nectar plants in herb-rich forests. Estimates are modelled median covers with 95% credible intervals in original and resurvey, and density plots show the posterior predictions of the medians.


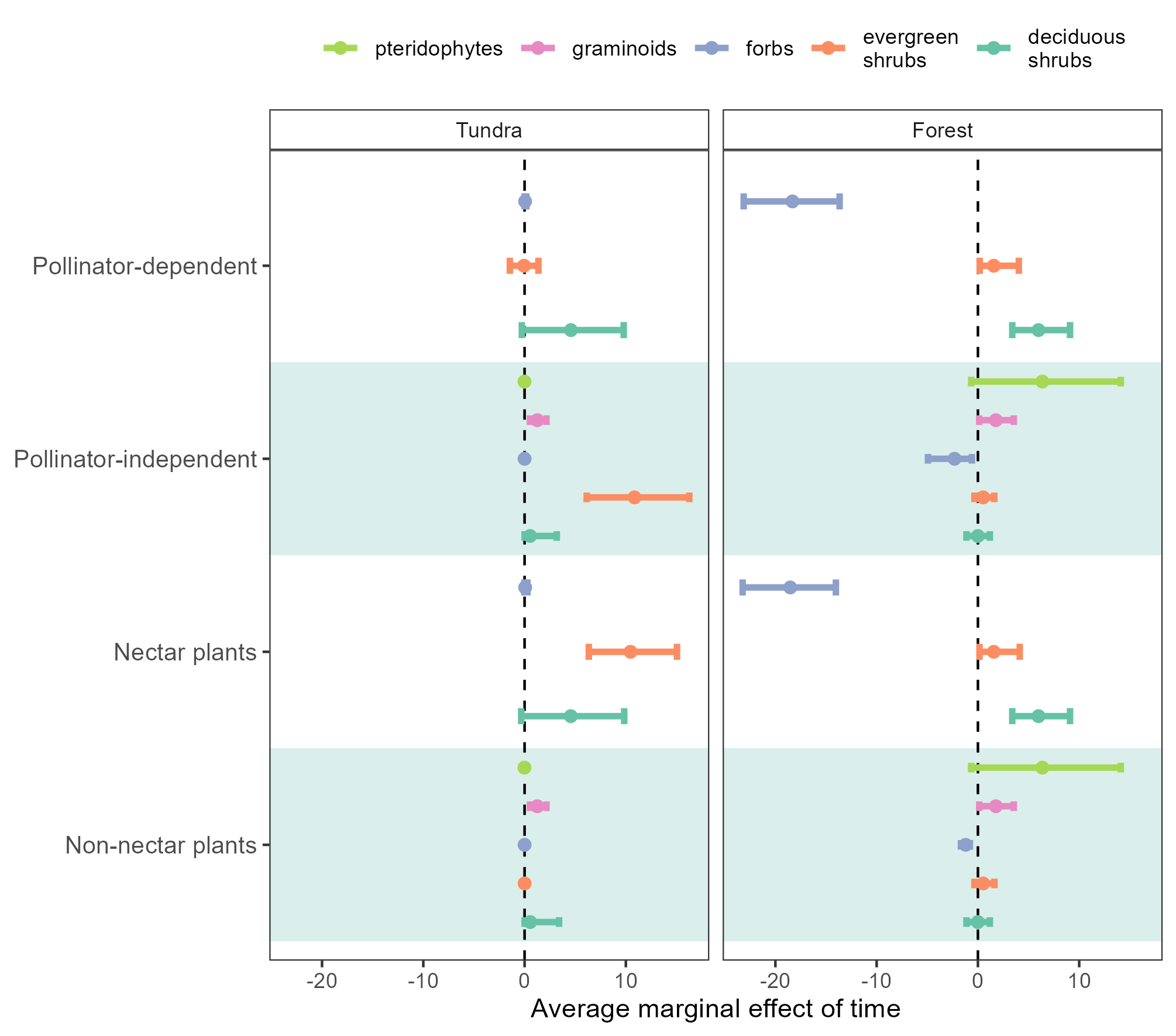
**Figure S4.** The estimated average marginal effect of time on the cover of plant growth forms in the tundra and herb-rich forests. The estimates are modelled medians with 95% credible intervals. Growth forms were assigned as pollinator-dependent, pollinator-independent plants, pollen-nectar and non-nectar plants before analysis based on species pollinator dependence and nectar production EIVs.

**Figure S5.** An additional post hoc model for the pollen-nectar plants of tundra showing both a) the estimated average marginal effect of time across all sites (median: 3.89; 95% CI: 0.75–7.50; in undisturbed (3.12; 95% CI: 0.05–6.18); disturbed sites (4.68; 95% CI: -1.03–10.70) on their absolute cover and b) their estimated average absolute cover in the original and resurvey when *Empetrum nigrum* was excluded from pollen-nectar plant cover. Estimates are modelled medians with 95% credible interval.


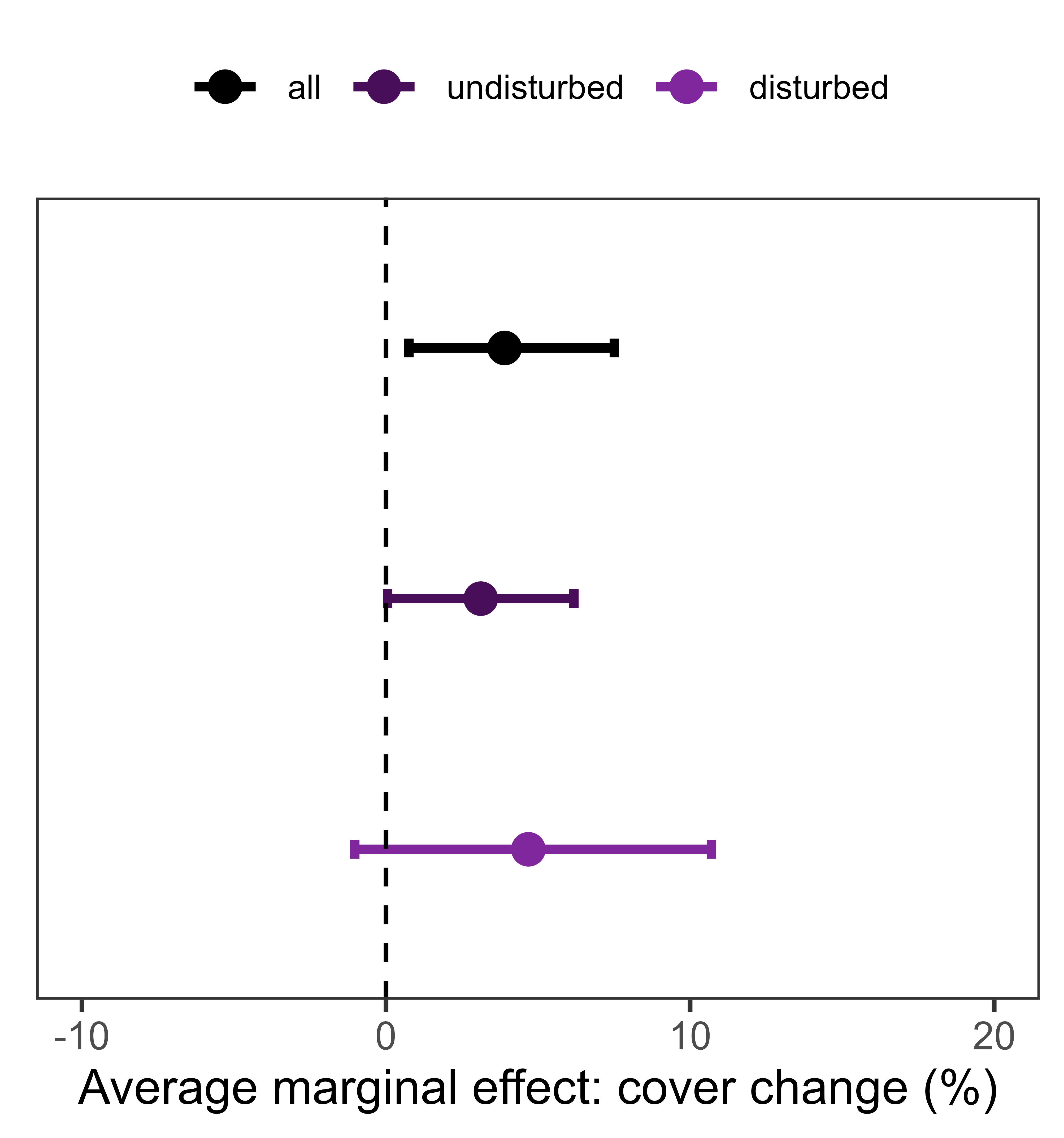

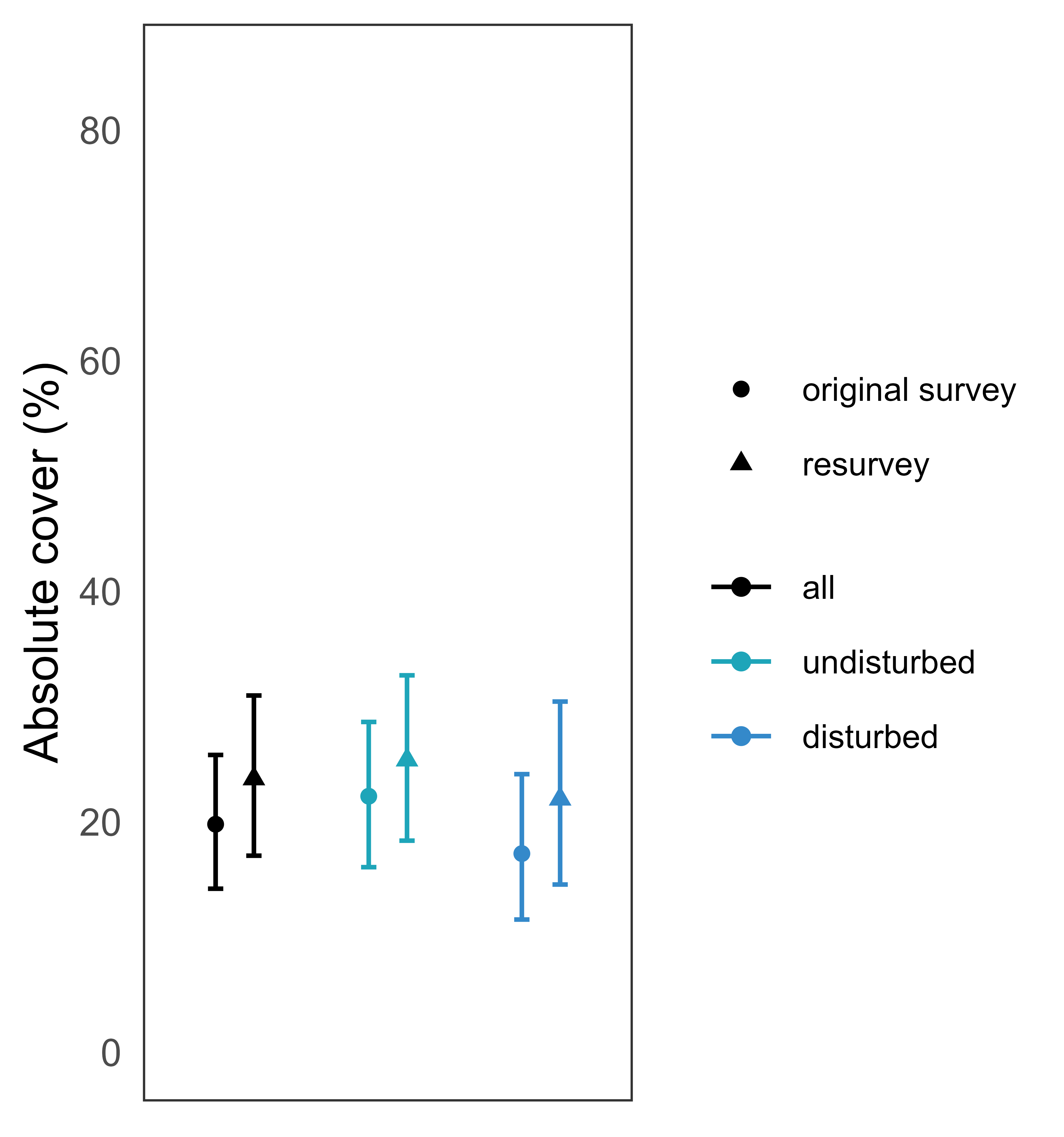


a)

b)
